# When it rains, it pours: Early treatment with tecovirimat of cardiac complications associated with monkeypox infection in a person with HIV and previously undiagnosed Lyme disease. A case report

**DOI:** 10.1016/j.heliyon.2023.e23965

**Published:** 2023-12-21

**Authors:** Filippo Lagi, Giuseppe Formica, Andrea Rostagno, Alessandro Milia, Silvia Pradella, Giulia Guazzini, Seble Tekle Kiros, Paola Corsi, Alessandro Bartoloni, Lorenzo Zammarchi, Filippo Pieralli

**Affiliations:** a Infectious and Tropical Diseases Unit, Careggi University Hospital, Florence, Italy; b Department of Clinical and Experimental Medicine, University of Florence, Florence, Italy; c Intermediate Care Unit, Careggi University Hospital, Florence, Italy; d Department of Radiology, Careggi University Hospital, Florence, Italy

**Keywords:** Monkeypox, Lyme, Tecovirimat, Myocarditis, Case report

## Abstract

Cardiac involvement, such as myocarditis and pericarditis, can be a severe complication of monkeypox virus (mpox) infection and could be related to other co-infections with cardiac involvement. Tecovirimat is an antiviral specifically designed to inhibit smallpox infection diffusion and approved by the FDA for other Orthopoxvirus infections; its efficacy in mpox-infected patients is not well established.

We present the case of a cardiac complication during mpox infection in a previously undiagnosed Lyme disease in a 42-year-old man living with HIV.

Two days after the typical maculopapular rash, the patient reported a rise in body temperature up to 39 °C, chest pain without irradiation, and shortness of breath. We found an increase in troponin level, a slight reduction in ejection fraction, and grade 2 AV block (Mobitz 1 and 2) with frequent sinus pauses (the longest of 10.1 s). Given the suspicion of myopericarditis with cardiac conduction system involvement, the patient was admitted to the Intermediate Care Unit for continuous monitoring and further evaluation. Treatment included Ibuprofen 600 mg every 12 hours (bid) and colchicine 1 mg once daily for anti-inflammatory purposes. Concomitantly, treatment with tecovirimat was started at 600 mg bid for a total of 14 days. Cardiac MRI with gadolinium showed mild interstitial edema and pericardial enhancement. However, despite the clinical and laboratory resolution of the acute phase, bradycardia with episodes of AV block persisted at follow-up, suggesting the possibility of an additional etiology. Thus, the patient was investigated for Lyme disease because high-degree AV block is the most common presentation of Lyme carditis. Serological results evidenced a previous *Borrelia burgdorferi* senso latu. We decided to start treatment with doxycycline 100 mg every 12h, even pending the uncertainty of the role of a previous Lyme disease in determining the cardiac rhythm disturbances. At the evaluation on day 44, the patient was systemically well, and after cardiologist consultation, pace-maker implantation was not deemed indicated.

This case underscores the importance of considering alternative causes of carditis when the clinical picture remains unclear or persists after the acute phase.

## 1 Background

Monkeypox virus (mpox) is an Orthopoxvirus genus, including variola virus, mainly prevalent in tropical forests of Central and Western Africa, responsible for a new outbreak in non-endemic regions with 86956 cases worldwide on the 17^th^ of April 2023 [1]. So far, around 957 cases have been reported in Italy since the start of the outbreak [1]. Mpox causes a systemic illness with fever, headache, muscle aches, back pain, low energy, and swollen lymph nodes, followed by the development of mucocutaneous manifestations, which may last for two to four weeks. The rash usually evolves into vesicular, pustular, and sometimes ulcerative lesions followed by the formation of dark crusts and can involve all body parts. During the current outbreak, mpox has shown a case fatality rate of around 0.04 %, a percentage much lower than 1–3% reported during previous outbreaks in West Africa over the past few decades [2]. Complications such as bronchopneumonia, encephalitis, myopericarditis, secondary bacterial infections, sepsis, and corneal or conjunctival lesions are unusual manifestations of mpox infection. The Food and Drug Administration (FDA) approved live, non-replicating vaccinia to prevent mpox infection, which is also available in Italy for not-immunized high-risk categories. The FDA approves tecovirimat (TPOXX or ST-246) for treating smallpox in adults and pediatric patients, but its efficacy against mpox has not been formally evaluated in clinical trials [3,4]. Tecovirimat is currently available for compassionate use to treat non-variola Orthopoxvirus infections, including mpox, in adults and children of all ages. Tecovirimat is a drug targeting the V061 gene that encodes for membrane protein p37, which is responsible for forming extracellular enveloped virions. Tecovirimat, through the inhibition of the formation of the VP37 envelope-wrapping protein, hastens the infection of the host's cells [5,6].

In September 2022, the AIDS Clinical Trials Group (ACTG) began the Study of Tecovirimat for Human Monkeypox Virus (STOMP), a randomized, placebo-controlled, double-blinded trial on the safety and efficacy of tecovirimat for acute mpox infection [7].

We present an unusual case of mpox-related cardiac complication in a person living with HIV with previously unknown Lyme disease and treated with tecovirimat. This case may increase clinician awareness regarding mpox complications, mainly to look for other causes of carditis. It also provides additional evidence to treat people living with HIV with tecovirimat.

## 2 Case presentation

### 2.1 Background

In this case study, we describe the complex clinical presentation of a 42-year-old Caucasian man who has sex with men living with HIV-1 for almost 12 years, regularly on follow-up at the HIV clinic, Careggi University Hospital. He had been successfully managed with antiretroviral therapy and had an undetectable viral load (defined as HIV-RNA <50 cp/mL) for more than ten years. He has been on tenofovir alafenamide/emtricitabine/rilpivirine (TAF/FTC/RPV) since 2018; it was his third line of antiretroviral therapy. The last CD4^+^ T cell count was 1184 × 10^6 cell/liter (CD4/CD8 ratio 1.9). Six years prior, he had received treatment for early latent syphilis, and aside from his HIV status, he had no significant medical history, risk factors for cardiovascular disease, or illicit drug use. He had also been fully vaccinated against SARS-CoV-2 but remained unvaccinated against smallpox.

### 2.2 Clinical presentation

**Fig. 1:**
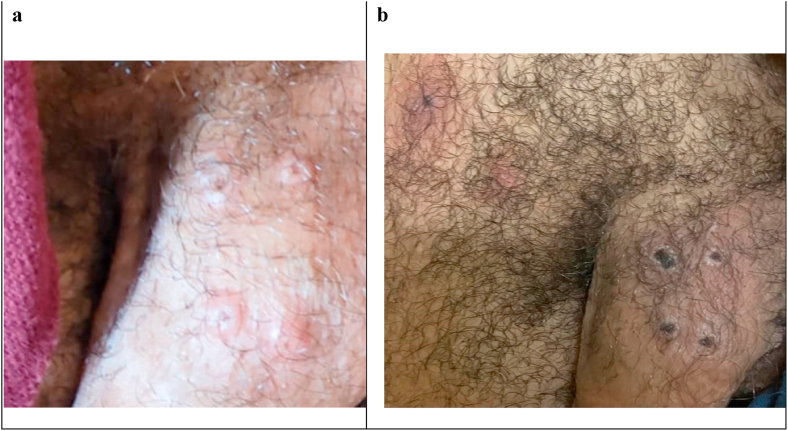
Clinical evolution in time of some skin lesions in the genital area of a patient living with HIV with monkeypox infection. a) First evaluation on day 2 from the onset of symptoms b) 6 days after the onset of symptoms and 4 days after the first evaluation.

In early September 2022, the patient sought medical evaluation due to a low-grade fever (37.5°) and the sudden appearance of skin lesions at the base of his penis. He reported recent sexual activity, including condomless oral sex and protected anal intercourse with multiple male partners. Physical examination revealed vesicular and umbilicated lesions on his genitals and small lymphadenomegaly in the groin area. Importantly, no oropharyngeal lesions were detected (Fig. 1).

### 2.3 Diagnostic evaluation

The PCR test for mpox-DNA on two skin lesions in the genital area turned positive. In contrast, a PCR test for mpox-DNA on the oropharyngeal swab was negative. Considering the absence of other symptoms, the patient was isolated at home according to the guidelines for managing cases published by the Italian Ministry of Health [8]. However, his condition worsened, with a gradual increase in body temperature up to 39 °C, chest pain, labored breathing, and shortness of breath. These symptoms prompted his referral to the emergency department (ED) for further analysis.

### 2.4 Hospital admission and diagnosis

Upon admission, the patient was hemodynamically stable and presented with continuous anterior chest pain exacerbated by deep breathing. The electrocardiogram (ECG) revealed sinus tachycardia with elevated PR segment in aVR lead and depressed in peripheral and thoracic leads. Echocardiography showed a slightly enlarged (left ventricle diastolic diameter of 59 mm) and a hypokinetic left ventricle with mildly reduced ejection fraction (EF 50 %). Notably, there was no evidence of pericardial effusion. Further imaging, including a chest CT angiography, ruled out pulmonary embolism and pneumonia.

The first value of high-sensitivity cardiac troponin (HScT) was 128,00 pg/ml (reference value < 14 pg/ml) and peaked at the second determination after 3 h at 142,00 pg/ml. Main clinical and laboratory findings at hospital admission are reported in Table 1. During initial ECG monitoring in the ED, persistent episodes of sinus pauses were recorded both during the day and the night time; the longest lasted 10.1 seconds and was associated with an atrioventricular block with two non-conducted P waves; other episodes of advanced AV block grade 2 Mobitz type 1 and 2 in a context of sinus bradycardia (heart rate 40–45, nadir 32 pulses/minute, while sleeping) were recorded.

**Table 1:** Clinical and laboratory findings of a patient living with HIV with monkeypox infection who presented to the emergency room complaining of chest pain and shortness of breath on the day of admission and a day 17th.

**Parameter**	**On admission**	**Day 17**	**Normal Values**
Blood pressure (mmHg)	125/80	130/80	–
Heart rate (Beats per minute)	70	72	–
Peripheral blood oxygen saturation (%)	99	98	–
Body temperature (°C)	36.0	36.2	–
Respiratory rate (breaths per minute)	16	14	–
C-Reactive Protein (mg/L)	72	9	<5
D-dimer (ng/ml)	1842	–	<500
Fibrinogen (mg/dl)	550	515	200–400
Troponin (pg/ml)	128.00	<3	<14
ALT (U/l)	41	31	10–50
Gamma-GT (U/l)	88	82	10–71
White blood count (×10^9/l)	9.22	5.62 × 10^9/l	4–10
Creatinine (mg/dl)	1.08	0.79	07–1.2
NT-ProBNP (pg/ml)	559	73	1–125

### 2.5 Diagnosis of myopericarditis

Given the suspicion of myopericarditis with cardiac conduction system involvement, the patient was admitted to the Intermediate Care Unit for continuous monitoring and further evaluation. Treatment included Ibuprofen 600 mg every 12 hours (bid) and colchicine 1 mg once daily for anti-inflammatory purposes. Concomitantly, treatment with tecovirimat was started at 600 mg bid. Although the patient remained stable during the day, nighttime monitoring revealed recurring AV blocks with pauses lasting more than 5 seconds, necessitating treatment with low-dose dopamine and standby external cardiac pacing. Atropine was administered when significant bradycardia occurred, although cardiac pacing was never required.

### 2.6 Exclusion of other infections

Multiple tests were conducted to exclude other potential infections. Nasal swabs for influenza A and B virus, as well as SARS-CoV-2, returned negative results. PCR for CMV, Epstein Barr virus, and the serology for CMV, EBV, Coxsackievirus, Echovirus, Parvovirus B19, and Herpes virus 1/2 were carried out: all of those tested negative excluding CMV and EBV serology resulting IgG positive and IgM negative. Syphilis serology documented previous but not active infection.

### 2.7 Progression and resolution

After nine days of hospitalization, the patient's left ventricular ejection fraction began to improve, eventually reaching normal values (EF 63 %).

**Fig. 2:**
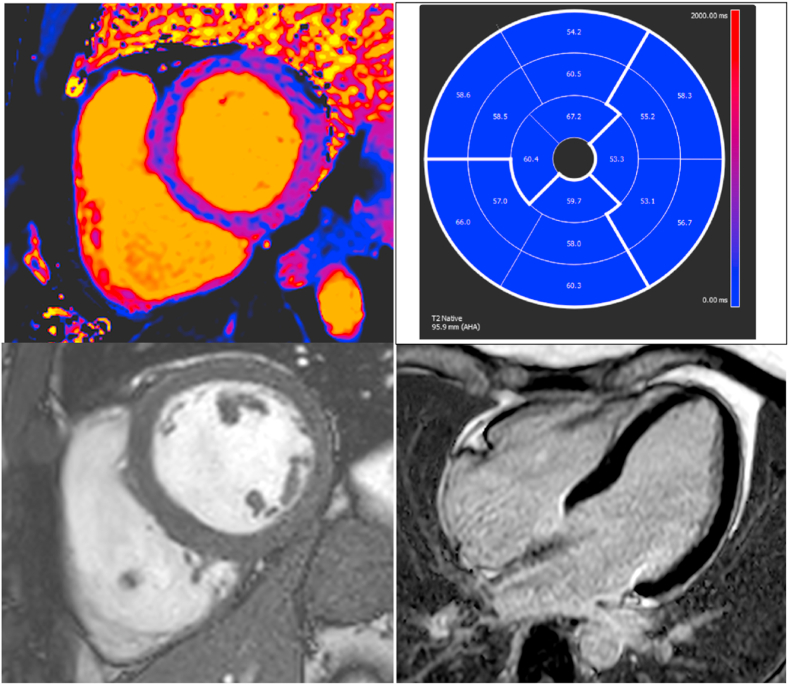
Cardiac magnetic resonance. A T1 color map, slight inhomogeneity and T1 value minimal increase, B T2-map values (slightly increased in all segments). C short axis cine Image D 4 chambers late gadolinium (LGE) PSIR image show no focal enhancement. (For interpretation of the references to color in this figure legend, the reader is referred to the Web version of this article.)

Cardiac magnetic resonance (MRI) with gadolinium on day 12 showed slightly increased global T1 and T2 mapping values, consistent with interstitial edema due to myocarditis; no signs of focal gadolinium enhancement or T2 mapping alterations were detected, while minimal hyperintensity of pericardium without effusion in LGE sequences was evident; global biventricular function (EF 57 %) was normal (Fig. 2).

Coronary CT angiography was normal, troponin levels normalized, and inflammatory chest pain wholly resolved. The patient was discharged on day 19 with a follow-up plan that included continuous ECG monitoring. He completed the 14 days of treatment with tecovirimat.

### 2.8 Discovery of Lyme disease

Subsequent 24-h ECG monitoring (day 26th) revealed nine asymptomatic episodes of AV block 2:1 type, especially during the nighttime with a maximum RR of 3,44s. Previous serologies were repeated, including *Borrelia* spp. antibodies, the latter to cover the possibility of Lyme disease with cardiac involvement. The enzyme immunoassay (LIAISON Borrelia; Diasorin; Italy) for *Borrelia burgdorferi* sensu lato complex (s.l.) tested IgG-positive and IgM-negative. Western immunoblot (Anti-Borrelia EUROLINE-RN-AT; EUROIMMUN Medizinische Labordiagnostika AG; Lübeck) confirmed the result of the EIA test (IgG-positive). When questioned, the patient did not recall any tick bite or suspected clinical symptoms of Lyme disease, even though he frequently trekked in the countryside. We analyzed a previously collected (June 2022) serum sample, obtaining the same result in EIA and WB tests.

### 2.9 Lyme disease treatment

The patient was started on doxycycline therapy 100 mg bid, which he completed over 28 days. After electrophysiologist revaluation, no indications at pace-maker implantation were placed. Clinical reasoning drove the decision to delay pace-maker implantation based on two key considerations. Firstly, the potential for reversible cardiac rhythm changes during acute cardiac inflammation. Secondly, the lack of urgent symptoms like hypotension or syncope necessitating immediate implantation. During a 44-day follow-up, he remained in excellent condition, with normal cardiac parameters and C-reactive protein values. A new 24-h ECG monitoring revealed persistent sinus rhythm with 3 episodes of 2nd degree AV block lasting less than 2 seconds during night sleep. These results indicated progressive resolution of the cardiac disease process, and a new ambulatory ECG monitoring was ordered after 6 months. The patient, regularly followed up at the HIV clinic, is wholly asymptomatic and in good clinical condition.

After reviewing the manuscript, the patient explicitly consented to the publication of the case provided that sensitive data were anonymized.

## 3 Discussion

This case report discusses a rare but severe cardiac complication of mpox infection in an individual living with HIV. Although the incidence is unknown, mpox life-threatening complications such as severe pneumonia, encephalitis, and myocarditis encephalitis seem rare. Myopericarditis is an inflammatory condition affecting both the cardiac muscle and the pericardium, and it can arise following various infectious or non-infectious triggers.

Typically, viral infections can lead to myopericarditis due to direct or indirect damage to cardiac structures. The severity of cardiac involvement can vary widely, ranging from acute fulminant presentation to milder cases with a benign course [9,10].

In this specific case, although the patient had a definite cardiac complication, clinical findings and cardiac MRI using the Lake Louise revised criteria do not fully support a diagnosis of myopericarditis [11]. Notably, there was a clear temporal association between the onset of cardiac alterations and the mpox infection. Nevertheless, mild myocarditis, which could lead to marginal changes in Cardiac MRI-derived T1 and T2 mapping, is not expected to reduce significantly the left ventricular ejection fraction (LVEF) (as it would require a more extensive myocardial injury), as observed in this case. Therefore, a definitive diagnosis of myocarditis as the single etiology of the myocardial compromise and dysfunction is challenging. Even the supposition of the occurrence of stress cardiomyopathy doesn't match the requirements for the diagnosis since regional wall motion abnormalities, which among other things, should extend beyond a single epicardial coronary artery distribution, were absent [12].

In this case, we could speculate that the initial and transient myocardial dysfunction, as indicated by a slight reduction in left ventricular ejection fraction (LVEF), elevation in cardiac troponin and NT-pro-BNP (Table 1), and suggestive cardiac MRI findings, could represent the initial phase of myocardial injury related to mpox infection, which might have been impeded by the early onset of tecovirimat, resulting in successful recovery of cardiac function. However, at a certain point along the way, we thought other causes should also be investigated. Despite the resolution of acute symptoms and laboratory findings, the patient continued to experience episodes of bradycardia with atrioventricular (AV) block 2:1, as detected by Holter ECG monitoring. The persistence of involvement of the cardiac conduction system is unusual in cases of acute myocarditis.

Recognizing that high-degree atrioventricular block is a common presentation of Lyme carditis, the patient underwent testing for Lyme disease. Serological results evidenced a previous *Borrelia burgdorferi* s.l. infection, leading to the decision to initiate antibiotic therapy. However, it was challenging to determine the precise role of previous Lyme disease in the cardiac abnormalities observed during the acute mpox infection.

The treatment with tecovirimat was well-tolerated by the patient, with only mild and self-limiting nausea noted shortly after starting the medication. Subsequent cardiac MRI performed five days after initiating tecovirimat therapy showed evidence of mild myocardial involvement with interstitial edema. It remains unclear whether tecovirimat played a role in reducing myocardial inflammation.

Limited clinical data exist on the use of tecovirimat for mpox. In an Italian study 19 out of 128 infected patients, of whom 7 people living with HIV (PLWH), were treated with antiviral therapy: 15 with tecovirimat and 4 with cidofovir based antiviral treatment. In tecovirimat treated patients, no side effects or alterations at blood tests were reported [13]. After reviewing the medical literature, myocarditis has been described in only a few cases of infections: the overall prognosis was good, with no mortality reported in all the described cases ([[14], [15], [16], [17], [18], [19]]). To the best of our knowledge, this is the first case of treatment, with a 14-day cycle of tecovirimat, for an infection complicated by myocarditis in a PLWH and previously undiagnosed Lyme disease.

Finally, like most patients with mild symptoms on presentation, our patient was in isolation at home. For those cases, we provided a dedicated email, daily check, and a phone number for communications. Although the complications are rare, maintaining regular contact with these patients and warning them to refer to ED in case of new symptoms, such as chest pain or neurological signs, is necessary.

## 4 Conclusions

Cardiac complications are possible severe complications of mpox infection with very few described cases in the literature. The report emphasizes the importance of considering multiple factors and potential co-infections in complex clinical cases like this. Tecovirimat, an antiviral developed for the treatment of smallpox, has uncertain efficacy against mpox infection. However, its prompt use is recommended in complicated forms and may have provided a clinical benefit in this case.

Finally, this report underscores the significance of regular follow-up and communication with patients with mpox infection, even in cases with initially mild symptoms, to ensure prompt evaluation and management of any new or concerning symptoms, particularly those related to the heart or nervous system.

## Ethic statement

After reviewing the manuscript, the patient explicitly gave written informed consent for the publication of the anonymized case details and images.

## Data availability statement

Data associated with this study has not been deposited into a publicly available repository. Data will be available upon request.

## CRediT authorship contribution statement

**Filippo Lagi:** Writing – review & editing, Writing – original draft, Formal analysis, Data curation, Conceptualization. **Giuseppe Formica:** Writing – review & editing, Writing – original draft, Formal analysis, Data curation, Conceptualization. **Andrea Rostagno:** Writing – review & editing, Investigation. **Alessandro Milia:** Writing – review & editing, Investigation. **Silvia Pradella:** Writing – review & editing, Supervision, Investigation. **Giulia Guazzini:** Writing – review & editing. **Seble Tekle Kiros:** Writing – review & editing, Investigation. **Paola Corsi:** Writing – review & editing. **Alessandro Bartoloni:** Writing – review & editing, Supervision. **Lorenzo Zammarchi:** Writing – review & editing, Supervision. **Filippo Pieralli:** Writing – review & editing, Writing – original draft, Supervision, Investigation.

## Declaration of competing interest

The authors declare the following financial interests/personal relationships which may be considered as potential competing interests:Professor L. Zammarchi currently serves as associate editor to the Infectious Diseases and Global Health section of the Journal If there are other authors, they declare that they have no known competing financial interests or personal relationships that could have appeared to influence the work reported in this paper.
